# Supplementary material for: Multi-Omics Elucidation of Flavor Characteristics in Compound Fermented Beverages Based on Flavoromics and Metabolomics
Source: Foods. 2025 Dec 1;14(23):4119. doi: 10.3390/foods14234119 (PMC12691716; doi:10.3390/foods14234119)
Supplement: Supplementary file 1 [file foods-14-04119-s001.zip › Supplementary materials.pdf]

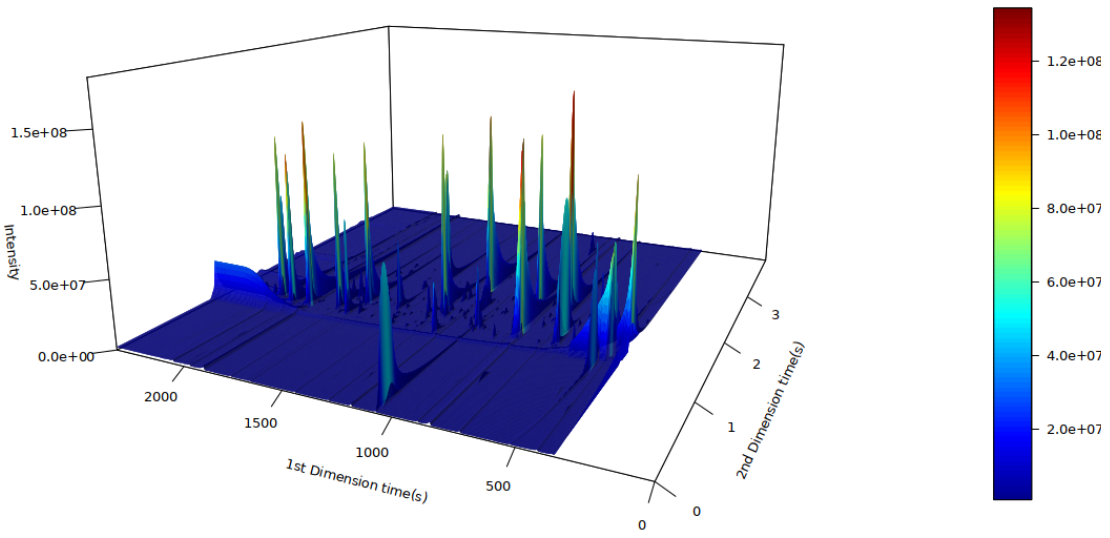

CW1(Mixing apples and grapes)

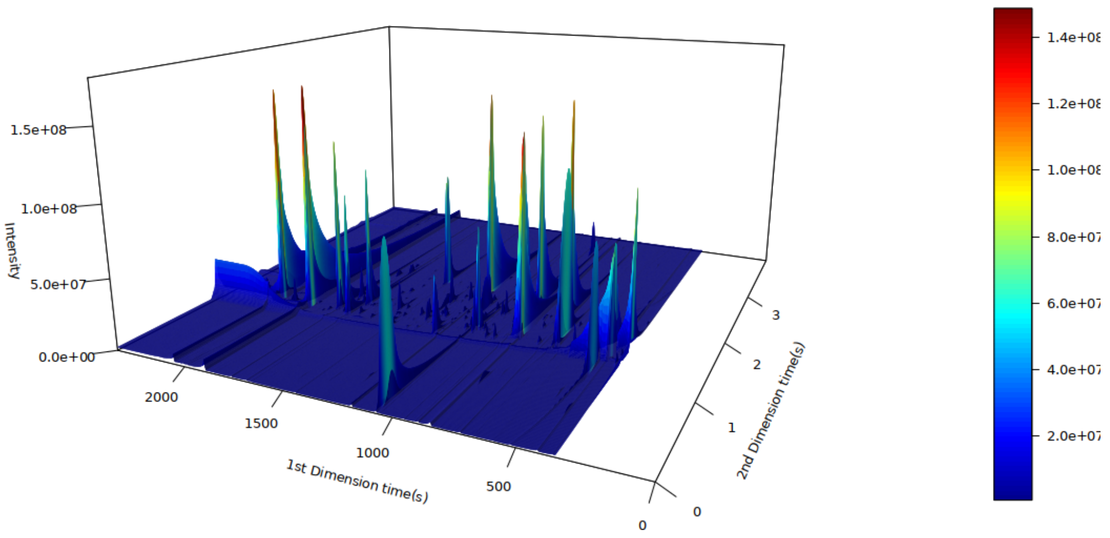

CW2(Mixing apples and grapes)

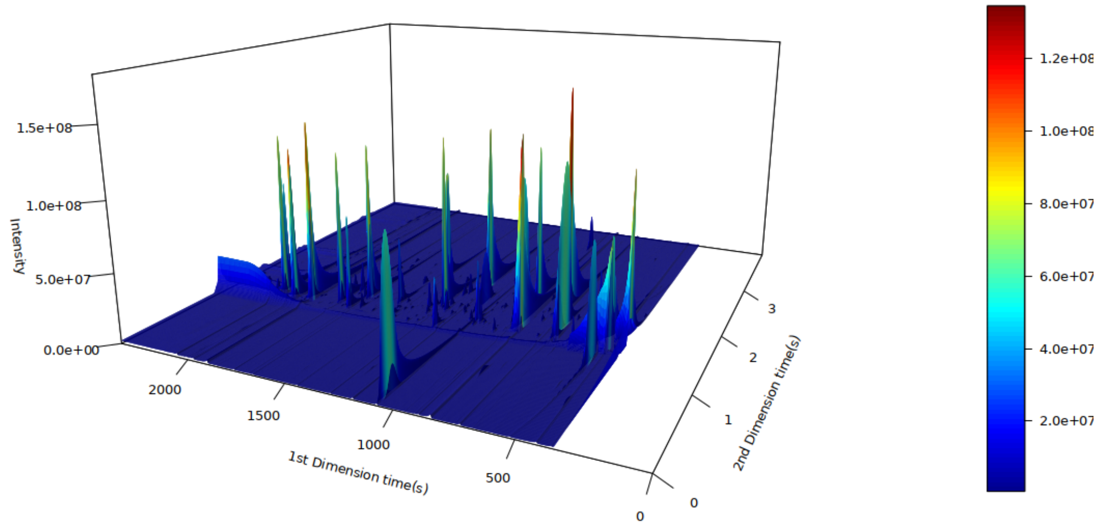

CW3(Mixing apples and grapes)

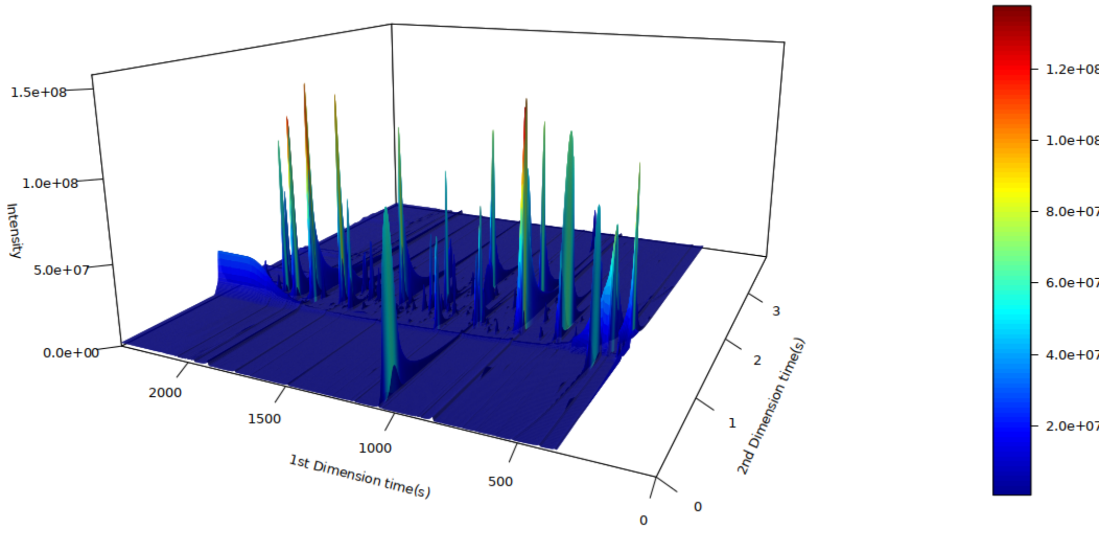

AW1(Apples)

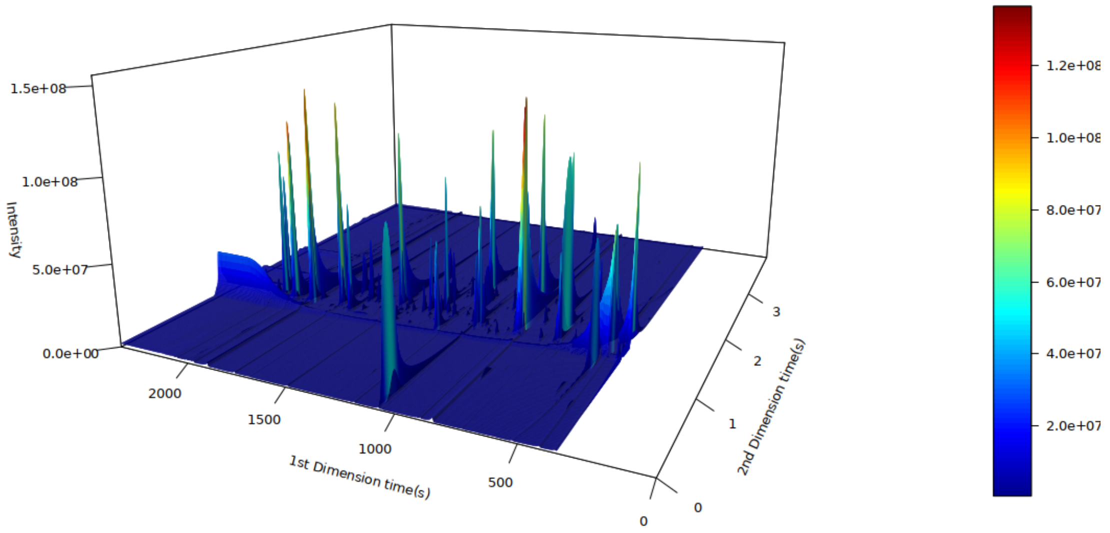

AW2(Apples)

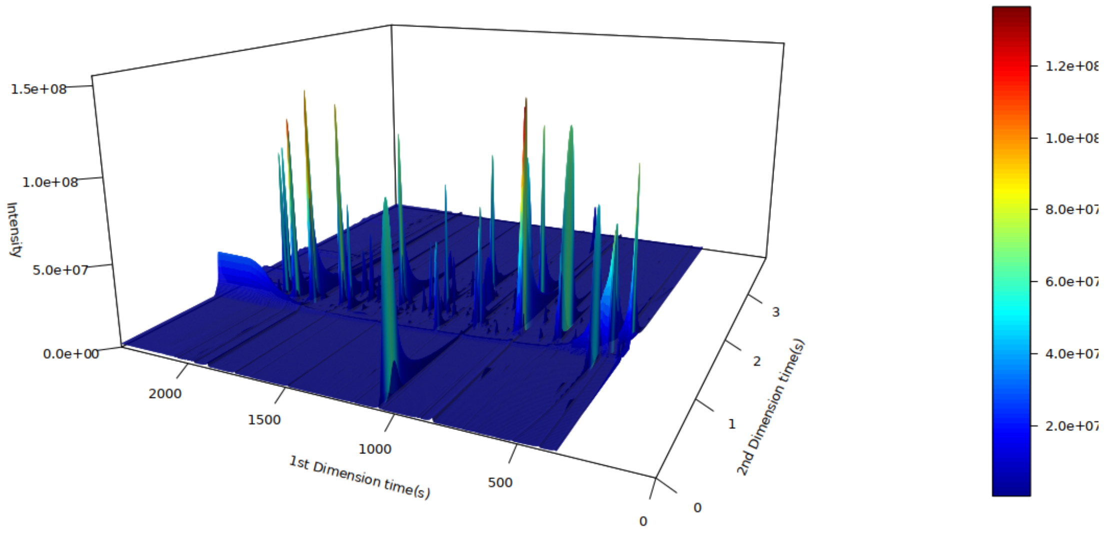

AW3(Apples)

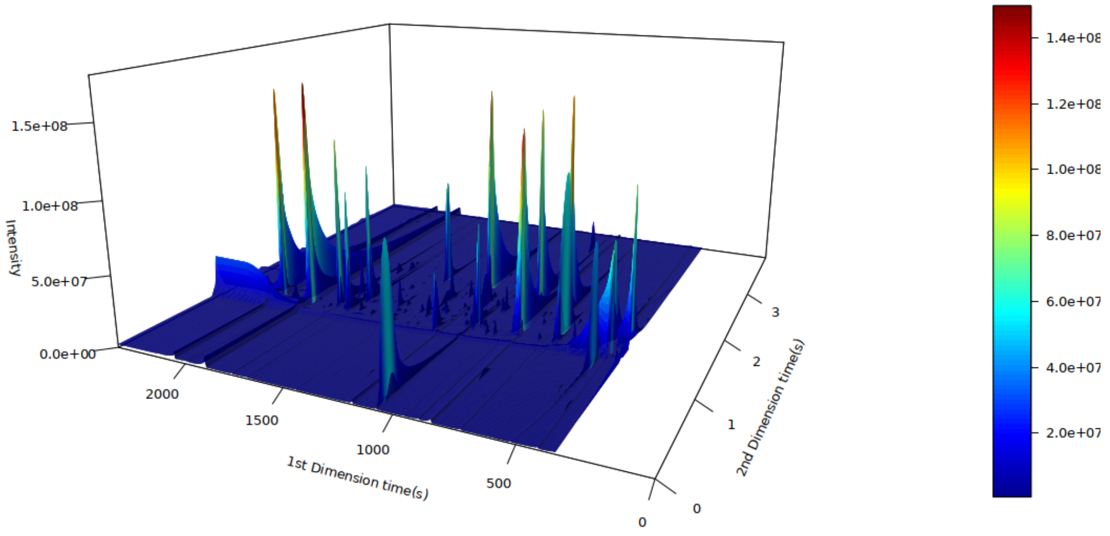

GW1(Grapes)

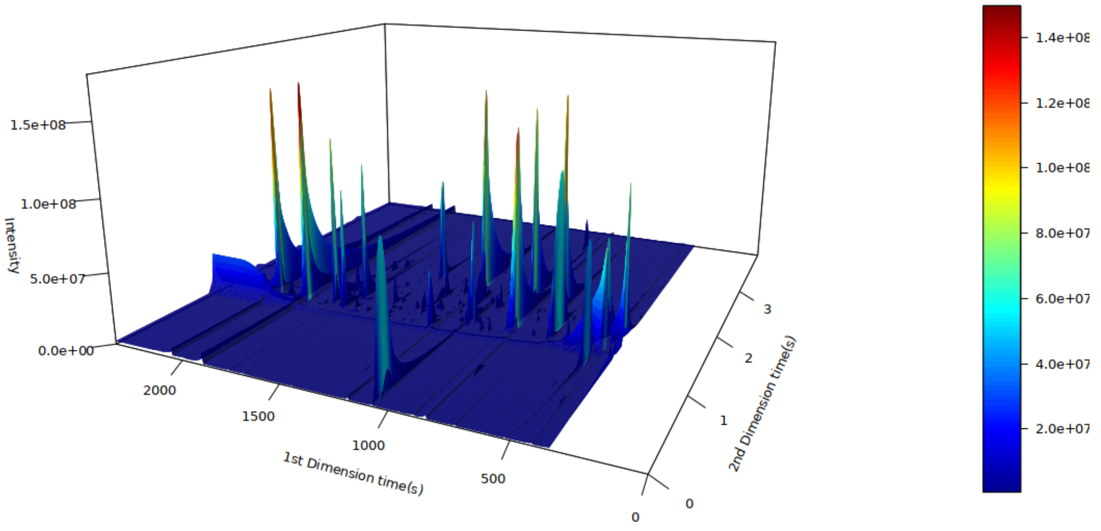

GW2(Grapes)

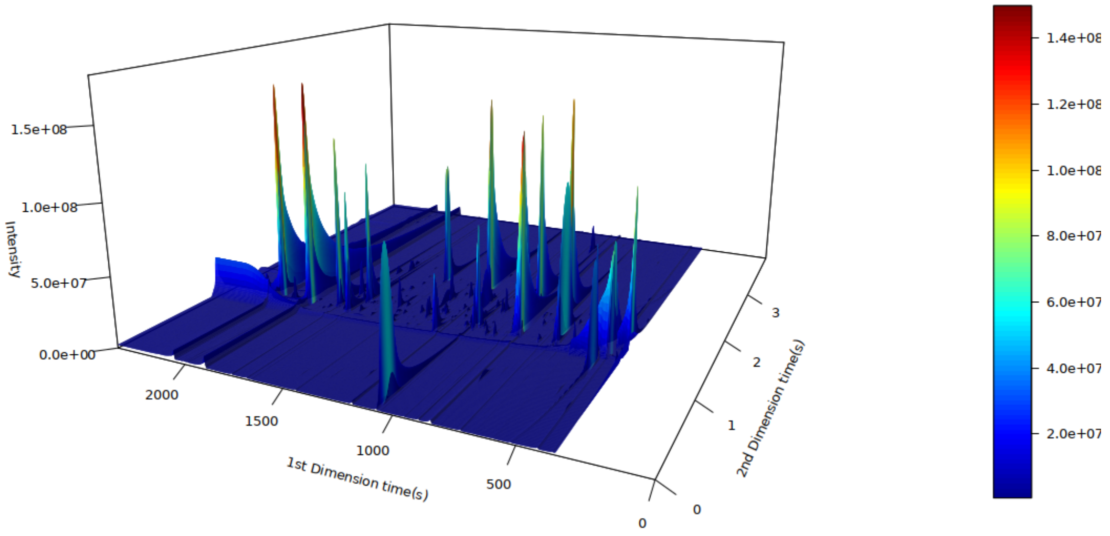

GW3(Grapes)

Figure S1. Composite fruit wine flavor ion spectrum, including apple and grape fermented beverages.

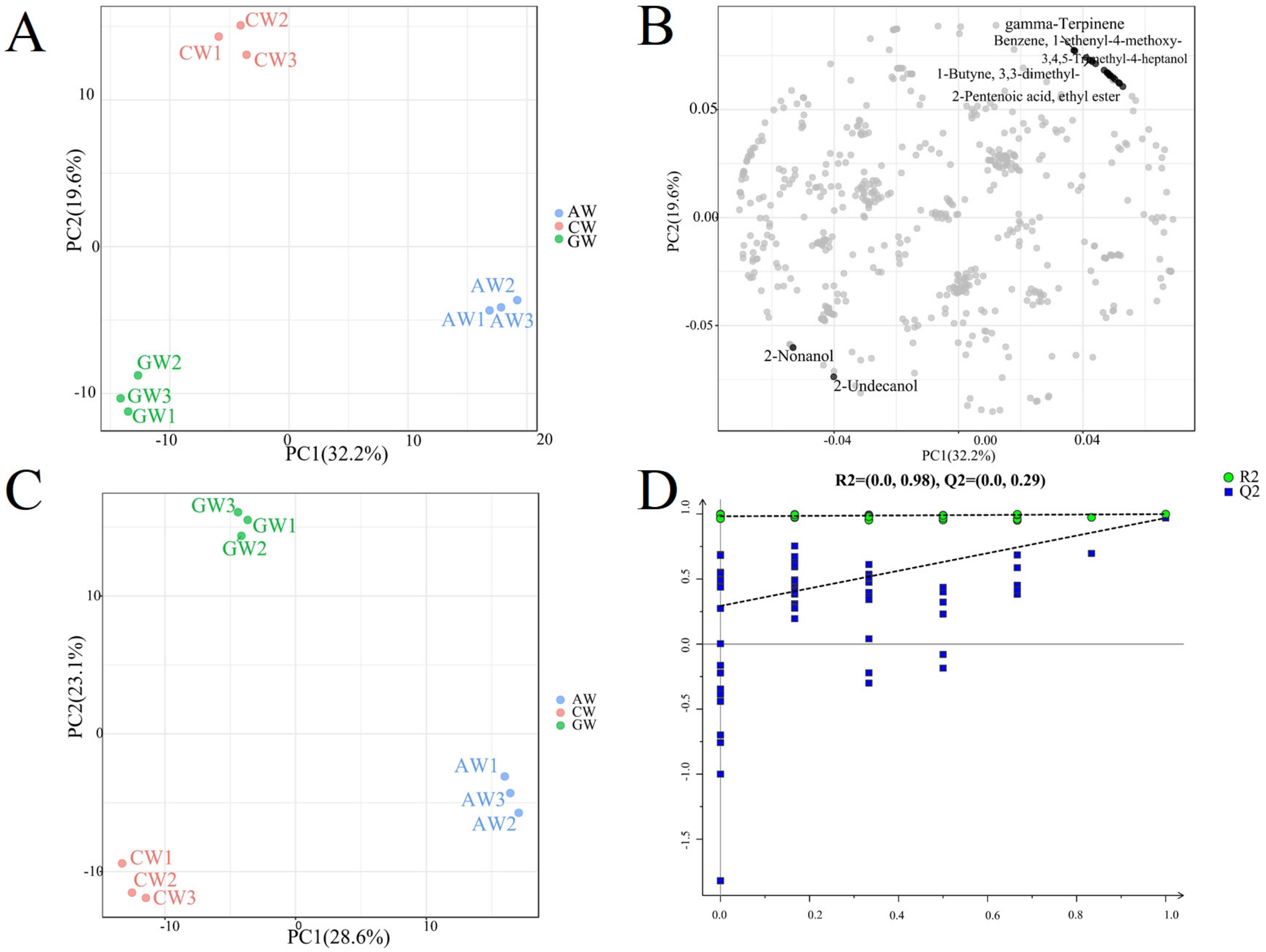

Figure S2. Statistical analysis of flavor products of three types of alcoholic beverages. (A)PCA。 (B) Principal component load distribution. (C)PLS-DA。 (D) Cross validation of the model.

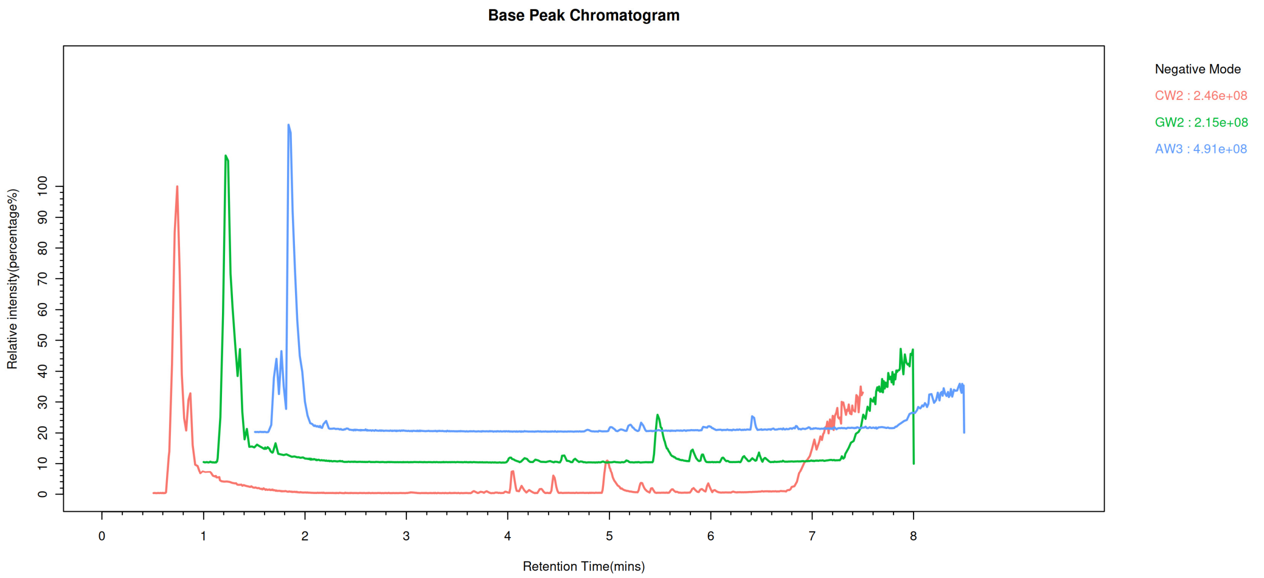

Typical sample peak chromatogram in positive ion mode (BPC)

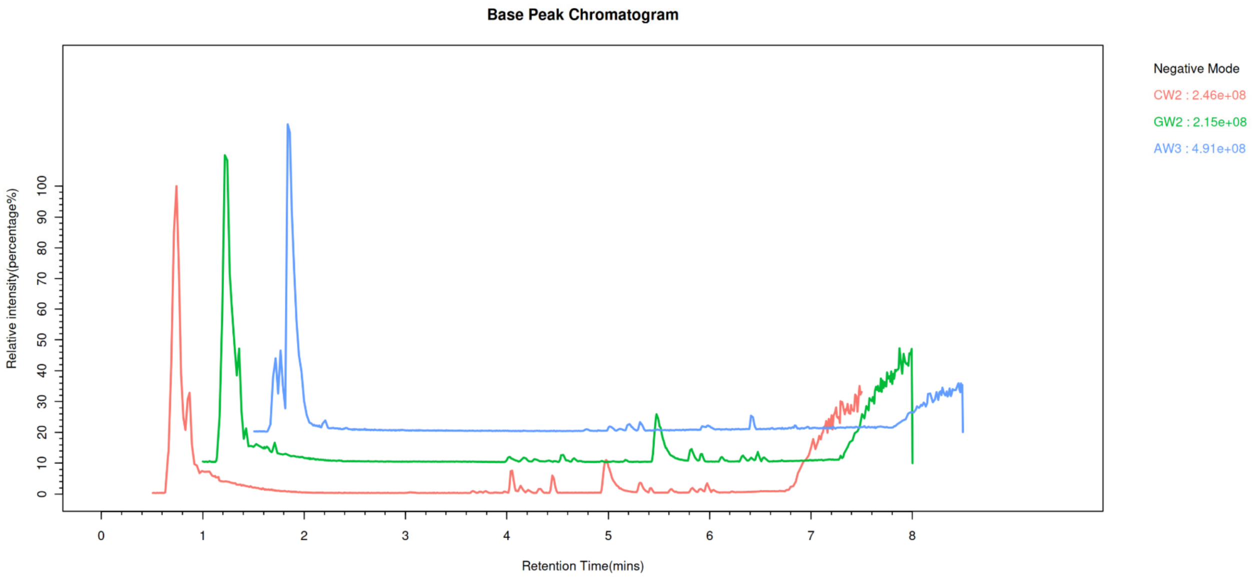

Typical sample peak chromatogram in negative ion mode (BPC)

Figure S3. Typical sample peak chromatogram of BPC ion mode

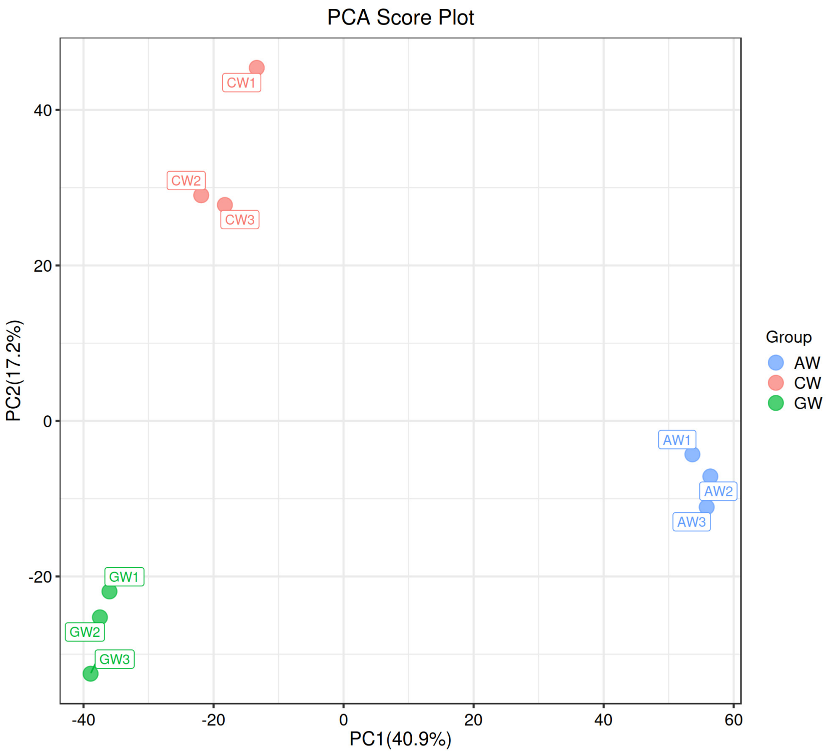

正离子模式 PCA

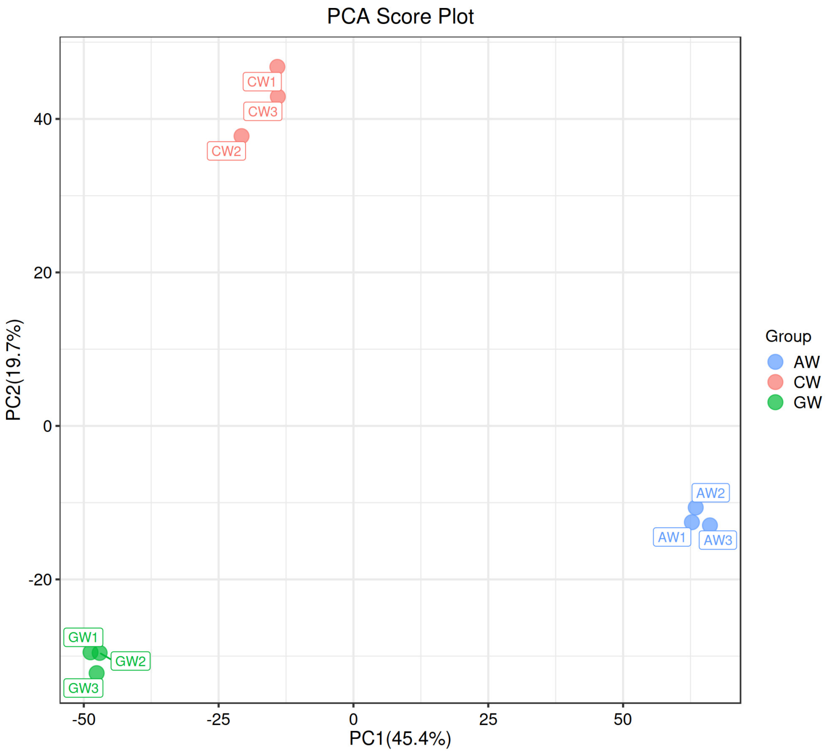

负离子模式 PCA

Figure S4. Perform PCA analysis (QC) on three types of fruit wine samples. AW

represents wine fermented from apples, GW represents wine fermented from grapes, and CW represents wine fermented from a mixture of grapes and apples.

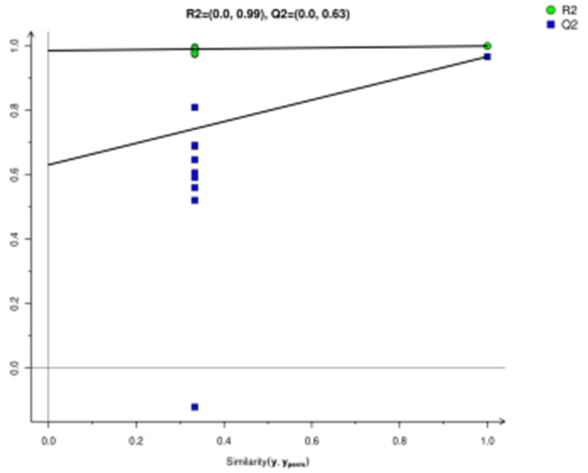

CWvsAW

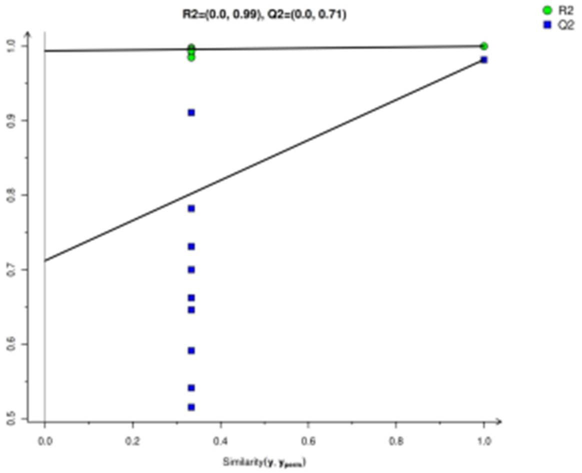

CWvsAW

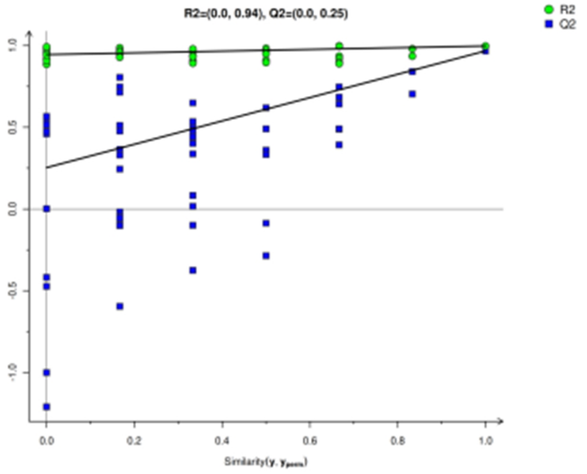

CW vs GW vs AW

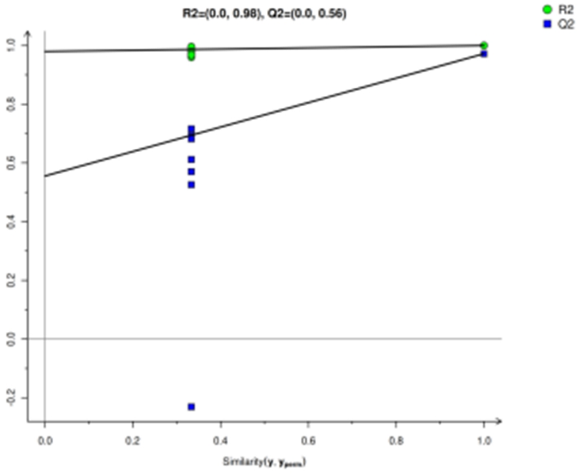

GWvsAW

Positive ion mode cross validation

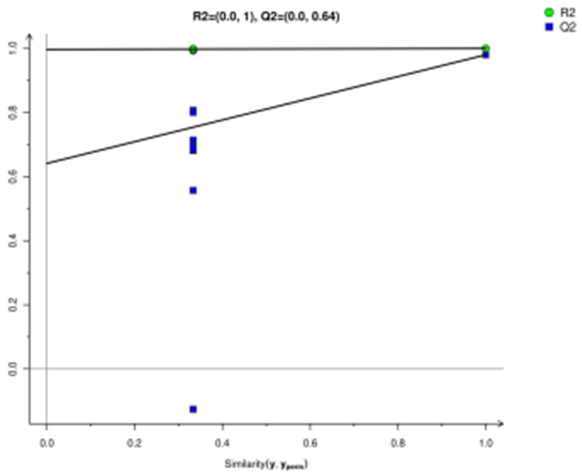

CWvsAW

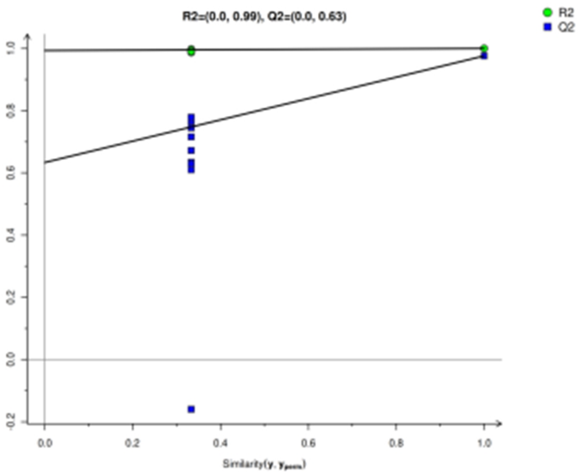

CWvsAW

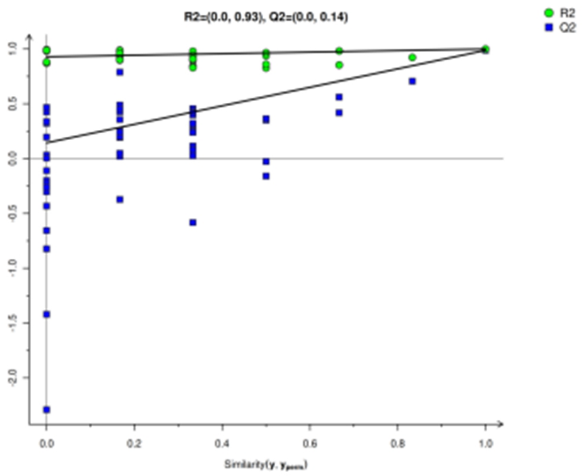

CW vs GW vs AW

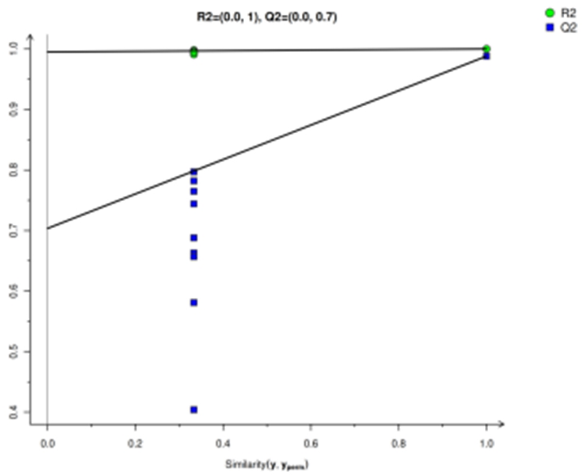

GWvsAW

Ion mode - cross validation

Figure S5. Dot plot of replacement test results for OPLS-DA model (n=200).AW represents wine fermented from apples, GW represents wine fermented from grapes, and CW represents wine fermented from a mixture of grapes and apples.

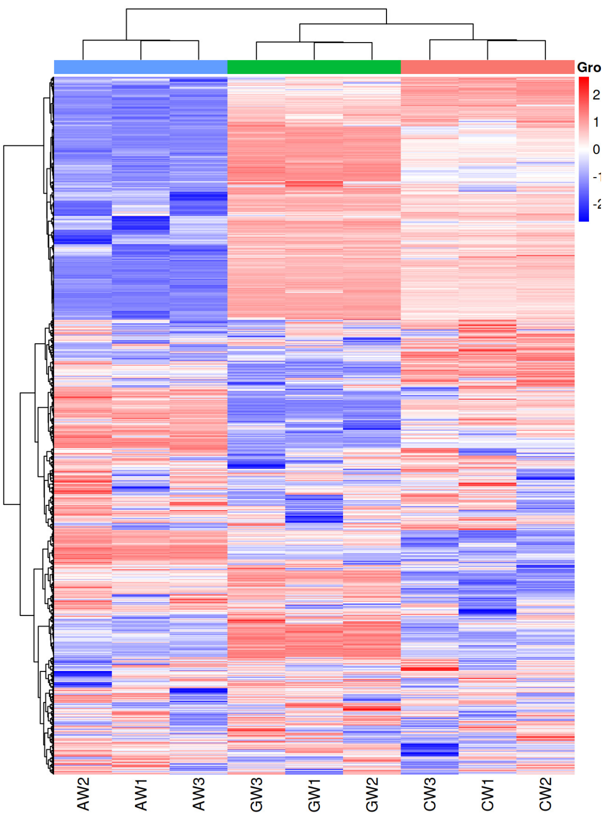

Negative ion mode

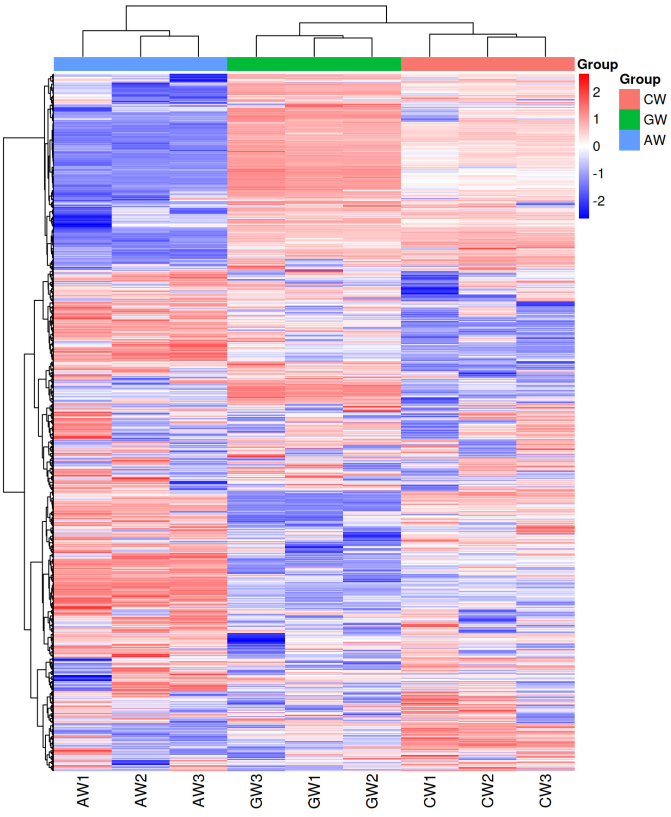

Positive ion mode

Figure S6. Ionic mode overall metabolite clustering heatmap.AW represents wine fermented from apples, GW represents wine fermented from grapes, and CW represents wine fermented from a mixture of grapes and apples.

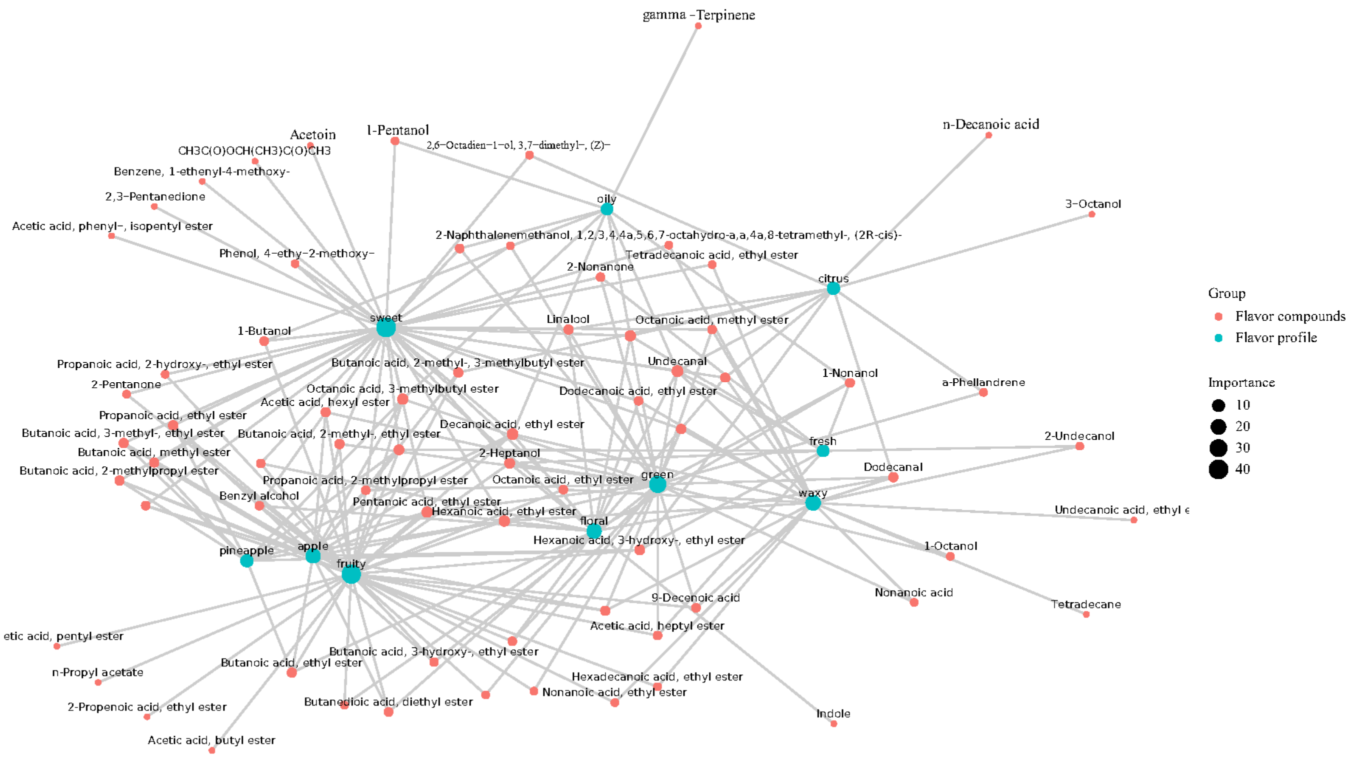

CWvsAW

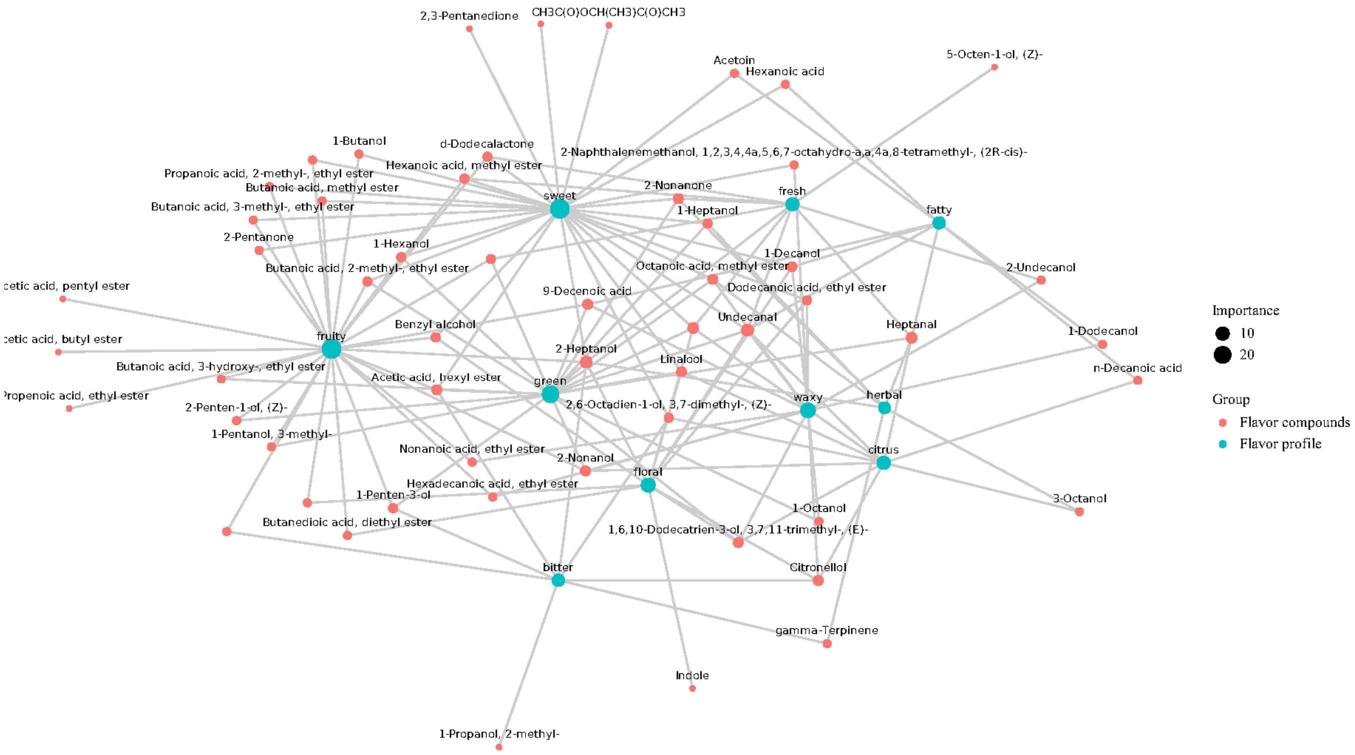

CWvsGW

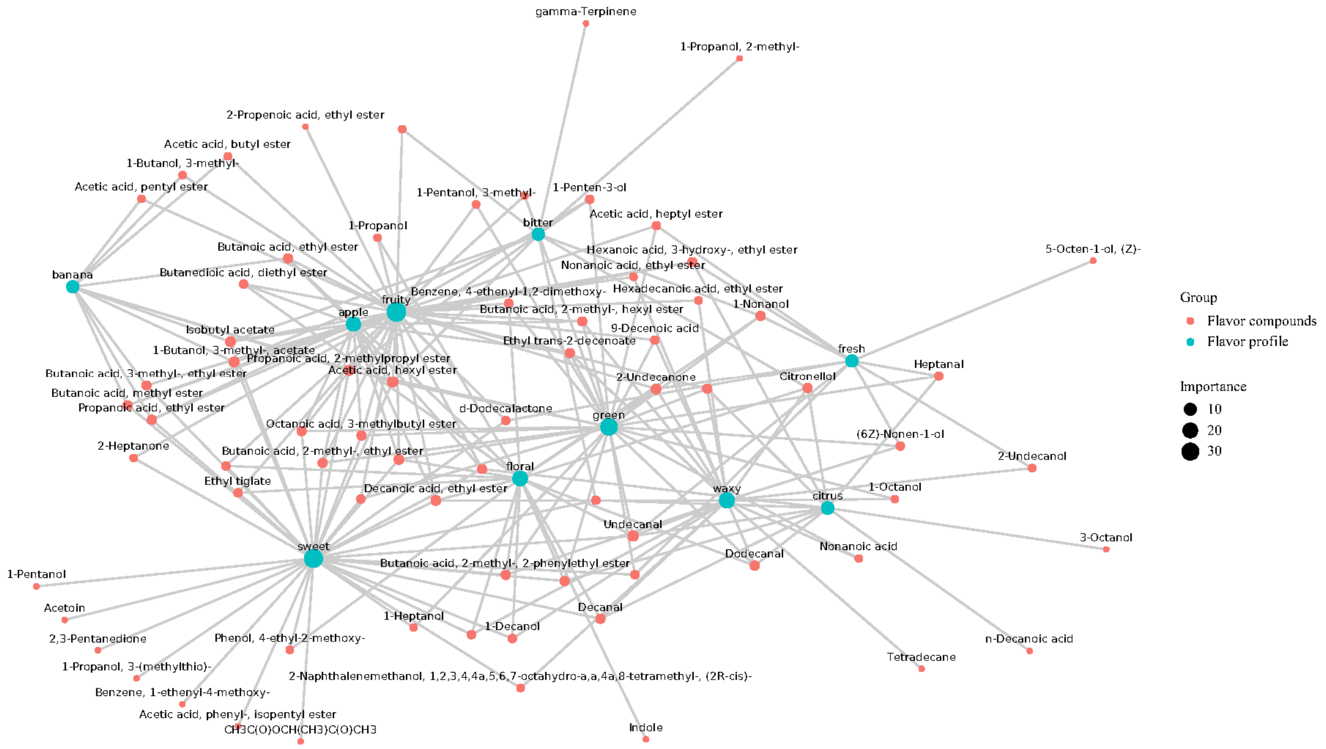

AWvsGW

Figure S7. Network diagram of the correlation between VOCs and sensory flavor attributes. Larger blue circles represent sensory attributes connected by more volatile organic compounds; The larger red circle indicates that volatile organic compounds contribute to more sensory attributes.

Table S1. Detailed Classification in Other Categories

Serial number	Classification	Quantity
1	Lignans, neolignans and related compounds	1
2	Nucleosides, nucleotides, and analogues	1
3	Organometallic compounds	1
4	Organophosphorus compounds	2
5	Homogeneous non-metal compounds	3
6	Phenylpropanoids and polyketides	4
7	Organosulfur compounds	10
8	Organic 1,3-dipolar compounds	11
9	Organohalogen compounds	19
10	Organic acids and derivatives	23
11	Furans and Pyrans	28
12	Sulfur-containing compounds	37
13	Nitrogen-containing compounds	54
14	Additional hydrocarbons not classified as major terpenes	136

15	Various heterocyclic compounds beyond the main categories	56

Table S2. PCA

Comparison	pre	R2X(cum)
GW vs AW	2	0.645
CW vs AW	2	0.638
CW vs GW	2	0.596
CW vs GW vs AW	2	0.518

Table S3. PLSDA

omparison	pre	R2X(cum)	R2Y(cum)	Q2(cum)
GW vs AW	2	0.636	1.000	0.973
CW vs AW	2	0.635	1.000	0.970
CW vs GW	2	0.587	1.000	0.963
CW vs GW vs AW	2	0.517	0.999	0.969

Table S4. Metabolite PCA

Comparison	pre	R2X(cum)
GW vs AW	2	0.620
CW vs AW	2	0.663

Comparison	pre	R2X(cum)
CW vs GW	2	0.693
CW vs GW vs AW	2	0.581

Table S5. Metabolite PLS-DA

omparison	pre	R2X(cum)	R2Y(cum)	Q2(cum)
GW vs AW	2	0.636	1.000	0.973
CW vs AW	2	0.635	1.000	0.970
CW vs GW	2	0.587	1.000	0.963
CW vs GW vs AW	2	0.517	0.999	0.969

Table S6. The Relative Odor Activity Values (ROAVs) of key differential volatile organic compounds (VOCs).

Name	Class	CAS	Formula	RangeofOdorMin	OdorCharacter	AW	GW	CW
1-Octen-3-ol	Alcohols	3391-86-4	C8H16O	6.12	Grassy, Fruity, Dusty, Oily	0.96	1.11	1.49
1-Propanol	Alcohols	71-23-8	C3H8O	53952.63	Fruity, Floral, Grassy	<0.1	0.1	<0.1
Benzyl alcohol	Alcohols	100-51-6	C7H8O	40927.16	Floral, Fruity, Sweet, Estery	<0.1	0.1	<0.1
2-Heptanol	Alcohols	543-49-7	C7H16O	1433.94	Peach Fragrance, Fusel Oil Smell, Fruit Fragrance, Floral Fragrance, Honey Fragrance	<0.1	0.01	0.1
1-Butanol	Alcohols	71-36-3	C4H10O	2733.35	Fruity	<0.1	<0.1	0.1
Octanal	Aldehydes	124-13-0	C8H16O	39.64	Grassy, Fruity	<0.1	<0.1	0.1
Heptanal	Aldehydes	111-71-7	C7H14O	409.76	Green Grass, Cucumber	0.1	<0.1	<0.1
3-methyl-Butanal	Aldehydes	590-86-3	C5H10O	16.51	Floral, Fruity	0.23	0.78	1.32
Phenol	Benzenoids	108-95-2	C6H6O	18909.34	Lyso Water, Like Glue, Ink	<0.1	<0.1	0.1

4-ethyl-Phenol	Benzenoids	123-07-9	C8H10O	617.68	Stinky Stables, Stinks Of Laishu Water, Stinks Of Cattle And Horse Pen	<0.1	<0.1	0.26
4-ethyl-2-methoxy-Phenol	Benzenoids	2785-89-9	C9H12O2	122.74	Melon, Fruit, Sweet, Floral, Smoky, Rubber Smell	<0.1	<0.1	1.48
3-methyl-Butanoic acid ethyl ester	Esters	108-64-5	C7H14O2	6.89	Apple Fragrance, Pineapple Fragrance, Banana Fragrance, Fruit Fragrance	0.96	0.77	1.26
Propanoic acid ethyl ester	Esters	105-37-3	C5H10O2	19019.33	Banana, Fruity	<0.1	<0.1	0.1
Ethyl Acetate	Esters	141-78-6	C4H8O2	32551.6	Pineapple, Apple, Fruit	0.07	0.09	0.08
2-methyl-Propanoic acid ethyl ester	Esters	97-62-1	C6H12O2	57.47	Sweet-Scented Osmanthus, Apple, Peach, Fruit	0.74	0.30	0.95
3-methyl-1-Butanol acetate	Esters	123-92-2	C7H14O2	93.93	Banana, Sweet, Apple, Fruit Candy	5.51	18.79	9.19
Pentanoic acid ethyl ester	Esters	539-82-2	C7H14O2	26.78	Peach, Fruity, Floral, Sweet	<0.1	<0.1	0.25
2-hydroxy-Propanoic acid ethyl ester	Esters	97-64-3	C5H10O3	128083.8	Sweet, Fruity, Grassy	<0.1	<0.1	0.1
Nonanoic acid ethyl ester	Esters	123-29-5	C11H22O2	3150.61	Ester, Honey, Fruit	0.1	<0.1	<0.1
Hexanoic acid ethyl ester	Esters	123-66-0	C8H16O2	55.33	Sweet, Fruity, Savory, Cucumber	18.34	8.09	27.4
Hexanoic acid propyl ester	Esters	626-77-7	C9H18O2	12783.77	Fruity, Ester, Laojiao, Pineapple, Sweet	<0.1	0.1	<0.1
Butanedioic acid diethyl ester	Esters	123-25-1	C8H14O4	353193.25	Fruity, Floral, Pollen	<0.1	<0.1	0.1
Decanoic acid ethyl ester	Esters	110-38-3	C12H24O2	1122.3	Pineapple, Fruity, Floral	1.02	0.94	0.20
Heptanoic acid ethyl ester	Esters	106-30-9	C9H18O2	13153.17	Floral, Fruity, Honey, Sweet	<0.1	<0.1	<0.1
Butanoic acid ethyl ester	Esters	105-54-4	C6H12O2	81.5	Apple, Pineapple, Fruity, Floral	5.18	5.20	5.14
Benzenepropanoic acid ethyl ester	Esters	2021-28-5	C11H14O2	125.21	Honey Pineapple Fragrance, Fruit Candy Fragrance, Honey Fragrance, Fruit Fragrance, Floral Fragrance	0.4	0.03	0.01
3-methyl-Butanoic acid	Lipids and lipid-like molecules	503-74-2	C5H10O2	1045.47	Sweat Smell, Sour Smell, Fat Smell	<0.1	0.2	<0.1
n-Decanoic acid	Lipids and lipid-like molecules	334-48-5	C10H20O2	13736.77	Goat Stink, Wine Stink, Rubber Stink, Paint Stink, Animal Stink	<0.1	0.17	<0.1

Octanoic acid	Lipids and lipid-like molecules	124-07-2	C8H16O2	2701.23	Fruity, Floral, Oily	0.30	0.80	0.64
Nonanoic acid	Lipids and lipid-like molecules	112-05-0	C9H18O2	3559.23	Fat Smell	<0.1	<0.1	0.2
Hexanoic acid	Lipids and lipid-like molecules	142-62-1	C6H12O2	2517.16	Sweat Smell, Animal Smell, Sour Smell, Sweet Smell, Fruit Smell	0.20	0.34	0.20
Heptanoic acid	Lipids and lipid-like molecules	111-14-8	C7H14O2	13821.32	Sour, Sweaty, Muddy, Musty	<0.1	0.1	0.1
Butanoic acid	Lipids and lipid-like molecules	107-92-6	C4H8O2	964.64	Sweat Odor, Sour Odor, Pit Mud Odor	0.3	0.01	0.06
Octanoic acid ethyl ester	Organoheterocyclic compounds	106-32-1	C10H20O2	12.87	Pear Fragrance, Lychee Fragrance, Fruit Fragrance, Sweet Fragrance, Lily Fragrance	100.	100	100

Note: Organic active small molecule (PubChem) database, available at: https://pubchem.ncbi.nlm.nih.gov/ . Flavordb database, website: https://cosylab.iiitd.edu.in/flavordb/

Table S7. The top 20 significant correlation links between key metabolites

serial number	CW vs GW vs AW		GW vs AW		CW vs AW		CW vs GW	
	Name	P.value	Name	P.value	Name	P.value	Name	P.value
1	(Z)-4-Decen-1-ol	0.0077	5-Octen-1-ol, (Z)-	0.0001	5-Hepten-2-ol, 6-methyl-	0.0001	5-Octen-1-ol, (Z)-	0.0001
2	2-Hexen-1-ol, (E)-	0.0042	(Z)-4-Decen-1-ol	0.0046	3-Buten-1-ol, 3-methyl-	0.0001	2-Penten-1-ol, (Z)-	0.0128
3	9-Decen-1-ol	0.0042	2-Hexen-1-ol, (E)-	0.0001	3-Methylpenta-1,4-diene-3-ol	0.0010	1-Penten-3-ol	0.0001
4	5-Hepten-1-ol, 2,6-dimethyl-	0.0209	5-Hepten-1-ol, 2,6-dimethyl-	0.0001	5-Hepten-1-ol, 2,6-dimethyl-	0.0001	1-Hydroxymethyl-2-methyl-1-cyclohexene	0.0001
5	2,3-Butanediol, [R-(R*,R*)]-	0.0043	1-Penten-3-ol	0.0048	1-Hydroxymethyl-2-methyl-1-cyclohexene	0.0032	Cyclohexanol, 4-(1,1-dimethylethyl)-, trans-	0.0001
6	1-Propanol, 2-methyl-	0.0191	1-Hydroxymethyl-2-methyl-1-cyclohexene	0.0001	Cyclohexanol, 4-(1,1-dimethylethyl)-, trans-	0.0010	1-Heptanol	0.0107
7	1,3-Propanediol, 2-ethyl-2-(hydroxymethyl)-	0.0230	Cyclohexanol, 4-(1,1-dimethylethyl)-, trans-	0.0001	1-Heptanol	0.0023	1-Propanol, 2-methyl-	0.0038

8	1-Propanol	0.0167	1-Heptanol	0.0014	1-Propanol, 2-methyl-	0.0359	Benzyl alcohol	0.0001
9	9-Decyn-1-ol	0.0146	2,3-Butanediol, [R-(R*,R*)]-	0.0015	Cyclohexanol, 4-(1,1-dimethylethyl) -, cis-	0.0010	1-Pentanol, 4-methyl-	0.0138
10	6-Methylheptane-1,6 -diol	0.0041	1-Butanol, 3-methyl-	0.0215	1-Nonanol	0.0013	3-Octanol	0.0082
11	3-Octanol	0.0319	1-Propanol, 2-methyl-	0.0442	1-Propanol	0.0057	3-Pentanol	0.0001
12	3-Pentanol	0.0320	1,5,7-Octatrien-3-ol, 3,7-dimethyl-	0.0038	Benzyl alcohol	0.0378	1-Pentanol, 3-methyl-	0.0015
13	2-Octanol	0.0041	1-Nonanol	0.0018	9-Decyn-1-ol	0.0002	3,4,5-Trimethyl-4-he ptanol	0.0001
14	2-Methyl-3-decanol	0.0082	Methanol-D4	0.0023	1-Pentanol, 4-methyl-	0.0434	1-Hexanol, 2-ethyl-	0.0001
15	2-Butyn-1-ol	0.0252	1-Propanol	0.0003	3-Octanol	0.0001	2-Undecanol	0.0001
16	3-Octanol, 2,3-dimethyl-	0.0220	Benzyl alcohol	0.0004	3-Pentanol	0.0000	2-Nonanol	0.0001
17	1-Pentanol	0.0333	(6Z)-Nonen-1-ol	0.0001	Ethyl 3-hydroxyoctanoate	0.0016	2-Heptanol	0.0001
18	1-Dodecanol	0.0094	9-Decyn-1-ol	0.0001	1-Pentanol, 3-methyl-	0.0442	1-Octanol	0.0009
19	(R)-(-)-2-Pentanol	0.0207	1-Pentanol, 4-methyl-	0.0082	3,4,5-Trimethyl-4-he ptanol	0.0001	1-Dodecanol	0.0073
20	4-Hexen-1-ol, acetate	0.0195	3-Octanol	0.0040	1-Hexanol, 2-ethyl-	0.0000	1-Hexanol	0.0004
